# A Treatise on the Chemical, Medicinal, and Physiological Properties of Creosote, Illustrated by Experiments, &c.

**Published:** 1836-07-01

**Authors:** 


A Treatise on the Chemical, Medicinal, and Physiological Properties of Creosote, illustrated by Experiments, &c. By *John Rose Cormack.* Octavo, pp. 154. Edinburgh and London, April, 1836.

This is a very creditable essay of Mr. Cormack's, and we augur well of its author. The work is divided into two parts and five chapters—in each of which Mr. C. evinces acumen, science, and research. Creosote is now at-

---

tracting much attention, and, therefore, this little volume will probably be popular. The most important chemical property of this substance is, doubtless, that of coagulating albumen, on which, no doubt, its antiseptic powers depend. The modus operandi of this coagulation is not exactly ascertained. Its preservation of dead animal matters from putrefaction, has led physicians to expect great things from it in the living body. We hope they will not be disappointed.

" The meats which are intended to be prepared, ought to be immersed in a solution of one part of creosote in a hundred of water. In this solution they should remain from twelve to forty-eight hours, according to their size, when they are to be dried either in the sun or before a fire, and afterwards set aside for six or eight days, at the end of which period they will be found to have acquired the appearance, consistence, smell, and taste of the finest smoked meat. Bullocks' tongues, mutton hams, haddocks, salmon, &c. &c. do well to be treated in this way. The tongues which I prepared in this manner equalled in delicacy of flavour the finest smoked rein-deer tongues so much prized by many epicures, and the haddocks were not inferior to those preserved with wood-smoke at the celebrated Finnan. Before leaving the subject of the domestic uses of creosote, it may be stated, that four drops of creosote added to a gallon of whiskey, impart to it a peculiar flavour, very similar to that well known by the name of the *Peat-reek.* Dealers have long been in the habit of giving the peat-reek taste to whiskey, with a view of making their article pass in the market as the much-prized product of the small Highland stills, by adding to it a minute quantity of the oil procured by the distillation of peat in close vessels. Since creosote has been introduced into this country, it has been substituted for the peat-oil, and is at present extensively used for this purpose, both by dealers and in some private families, as I have occasion to know." 19.

Mr. C. thinks there can be no doubt that bodies, embalmed by means of creosote, would resist the tooth of time as long as the best Egyptian mummies. He dedicates a chapter to this subject, which, however, we shall pass over.

In the 3d chapter of the second part, Mr. C. takes up the physiological effects of creosote, and details several experiments on animals with this substance. The tenor of these experiments proves that our author is a " DESTRUCTIVE," rather than a " CONSERVATIVE."

In the first and second experiments, pure creosote, in quantities of ʒj. in one, and 25 drops in the other, were injected into the jugular and femoral veins of dogs. The action of the heart was suddenly suspended, and the animal died. Other experiments were made, in which creosote, in poisonous doses, was injected into the stomach.

" Its first deleterious action is the production of a powerfully sedative effect upon the heart, and from the rapidity with which this is manifested, it may be said (without using the expression at all in a figurative manner), almost instantaneously to paralyze the vital energies of that organ. In some instances, hurried and sonorous respiration goes on for more than a minute after the heart ceases to beat." 70.

In other experiments, creosote was thrown into the arteries—the injurious effects being much milder than when it was injected into the veins. If, indeed, the dose be not considerable, the animal experiences but little inconvenience.

From various experiments performed by our author, it would appear that the poisonous effects of creosote and prussic acid are strikingly similar. It is also a curious fact that, by large doses of both these substances, vomiting is excited—while from the same doses, when given with the view of checking vomiting, no good results. Small doses appear to appease vomiting.

"The manner of treating a case of poisoning with creosote ought evidently to be similar to that adopted for the counteraction of the effects of prussic acid, namely, the exhibition of ammonia, chlorine, and other stimuli."

"In medicine, creosote may be used with great advantage as a sedative or anodyne. To produce such effects it is given in diseases of the heart, pulmonary complaints, vomiting, and to allay the pain of cancer, &c. A patient under Dr. Shortt's care in the Royal Infirmary, afflicted with cancer of the stomach, derived relief from pain in ten minutes after taking a dose of fifteen drops. When its sedative or anodyne action is wished speedily, the object is best attained by inhaling its vapours. When creosote is persevered with, in small and gradually increased doses, it acts as a tonic. In general it does not affect the bowels; but in two cases in which Dr. Shortt pushed its use to a considerable extent, diarrhœa, and in one of the instances, decided dysentery were produced." 93.

The effects of creosote on the urinary organs are very capricious. A drop of pure creosote, introduced into the hollow of a tooth, is, our author thinks, the best remedy for tooth-ache that has yet been discovered. The creosote should be introduced by means of a camel's-hair pencil, and some cotton afterwards put into the cavity, saturated with pure creosote. A solution of three or four drops of creosote to the ounce of water is recommended, in burns and scalds, by Berthelot. It is said to arrest hæmorrhage, even from large vessels. It is probable that it might prove a useful styptic where leech-bites are troublesome. In scrofulous, aphthous, phagedenic, and venereal ulcers, it has been found beneficial. Many continental surgeons assert that creosote has proved eminently useful in cancer. M. Breschet bears testimony to this point.

"M. Téalier applied a saturated solution of creosote in water to an open cancer, situated on the breast of a woman, who was suffering most excruciating pain from it; and it was with a view of alleviating this that he employed the creosote. The result is interesting. No sooner had the solution come in contact with the ulcerated surface, than the patient complained of an acute burning pain in the sore, shooting through the right side of the chest, and extending from the head to the very tips of the toes. This continued for an hour, after which the pain entirely ceased, and the patient enjoyed uninterrupted sleep for ten hours." 114.

The same author used the creosote in ulcers of the cervix uteri. The cases, however, are not very satisfactory. The author himself applied pure creosote to lupus of the nose, of three years' standing. The patient expressed no uneasiness, but the disease was ultimately rendered worse, and the remedy was abandoned. We refer to the various statements of Dr. Elliotson and others for further particulars.

